# The Value of Neutrophil/Lymphocyte Ratio Combined with Red Blood Cell Distribution Width in Evaluating the Prognosis of Emergency Patients with Sepsis

**DOI:** 10.1155/2022/1673572

**Published:** 2022-11-10

**Authors:** Maosheng Lin, Louwei Zhang, Xuhua Tang, Yejiang Tang

**Affiliations:** Department of Emergency Medicine, Zhuji Affiliated Hospital of Wenzhou Medical University, Zhuji, Zhejiang 311800, China

## Abstract

Sepsis is a dysfunction of various organs caused by a dysfunctional host response induced by infection. In recent years, the mortality rate of sepsis patients, especially the mortality rate of septic shock patients still remains high. Due to the complexity and heterogeneity of sepsis, there is currently a lack of clinical biomarkers that can be widely used for the early assessment of sepsis. In order to find more concise and accurate biomarkers for timely and adequate intervention in sepsis, we explored the value of neutrophil/lymphocyte ratio (NLR) combined with red blood cell distribution width (RDW) in assessing the prognosis of emergency sepsis patients. The results showed that NLR and RDW were closely related to the prognosis of emergency sepsis patients. The combination of the two can evaluate the prognosis of patients with emergency sepsis, which deserves close attention from clinicians.

## 1. Introduction

Sepsis is a common systemic inflammatory disease. Some studies have pointed out that sepsis can be caused by an infection in any part of the body, and most patients have clinical manifestations such as fever, shortness of breath, tachycardia, and increased peripheral blood leukocytes [1, 2]. At present, the clinical use of bundled and target-guided treatment programs for patients with sepsis can delay the development of patients' disease, but there are still some emergency sepsis patients with high mortality. Therefore, early identification and evaluation of the prognosis of emergency sepsis patients Influencing factors are the current clinical concerns [3]. At present, only SOFA, A-Pache II score, and some serum markers are used to predict and evaluate the progression of sepsis, but due to liver dysfunction and the use of other specific drugs, the above indicators still have certain limitations. Red blood cell distribution width (RDW), a simple and inexpensive parameter reflecting the degree of red blood cell volume heterogeneity, has traditionally been used for differential diagnosis of anemia in laboratory hematology. Recently, RDW has attracted extensive attention as a prognostic marker for various diseases such as acute myocardial infarction, heart failure, autoimmune diseases, and liver disease. A recent analysis has shown that the neutrophil-to-lymphocyte ratio (NLR) is a marker for the assessment of systemic inflammatory response, and a recent analysis has shown that NLR levels increase with the progression of sepsis in patients with sepsis [4]. There are many clinical reports on the influencing factors of the prognosis of emergency sepsis patients, and significant results have been achieved. However, there are few clinical studies on the effect of NLR combined with RDW on the prognosis of emergency sepsis patients [5, 6]. Therefore, the purpose of this study was to analyze the predictive impact of NLR and RDW on the prognosis of emergency sepsis patients in order to explore whether they can be used as a prognostic index in sepsis patients. The research is reported as follows.

## 2. Materials and Methods

### 2.1. General Information

A retrospective analysis of 63 patients with acute sepsis admitted to our hospital from May 2020 to May 2022 was included as the research object. Among the 63 patients, 36 were males and 27 were females; the age ranged from 18 to 70 years, with an average age of (43.82 ± 4.16) years. This study has been approved by the hospital ethics committee, and all patients are informed and sign the relevant consent form.

### 2.2. Inclusion Criteria

(1) Those who meet the diagnostic criteria for emergency sepsis [7]; (2) those aged ≥18 years; (3) those who have been admitted to the hospital for ≥24 hours; (4) patients without mental illness or family history; and (5) those who have complete clinical data.

### 2.3. Exclusion Criteria

(1) Patients with other end-stage chronic diseases; (2) patients with other infectious diseases, immune system diseases, and blood system diseases; (3) patients with abnormal coagulation function; (4) patients with hemorrhagic shock; (5) patients with a history of erythrocyte suspension infusion in the past 7 days; (6) patients with a history of erythropoietin, cyclosporine, and other drug treatments in the past 7 days; (7) patients with a history of chemotherapy; (8) patients combined with cardiogenic shock, acute myocardial infarction, and other emergencies.

### 2.4. Research Methods

The general information of all patients was collected, including the patients' age, gender, body mass index (BMI), underlying diseases, and sepsis-related organ failure assessment (SOFA). record the level of laboratory indicators, including high density lipoprotein-cholesterol (HDL-C), total cholesterol (TC), low density lipoprotein-cholesterol (LDL-C), triglyceride (TG), activated partial thromboplastin time (APTT), D-dimer (DD), thromboplastin time (TT), prothrombin time (PT), hematocrit (Hct), hemoglobin (HB), platelet (PLT), RDW, and NLR.

Among them, the weight and height of the patients were collected, and calculate the BMI value according to the formula (BMI = weight/height^2^) [8]. And the survival of the patients within 30 days was counted. In addition, the levels of Hct, HB, PLT, NLR, and RDW were measured by the BS-20S automatic blood cell analyzer (Mindray). LDL-C, HDL-C, TC, and TG levels were measured directly. The Dd were determined by nephelometry; the levels of APTT, platinum, and TT were determined by coagulation. All the kits were provided by Shanghai Kaibo Biology Co., Ltd. The SOFA score included six items including respiratory system (PaO_2_/FiO_2_ oxygenation index) (mmHg), platelet count, bilirubin, circulatory system function, GCS score, and renal function. When the daily changes of SOFA score ≥2, it could be considered that the infected patient had an acute change in organ failure. The higher the score is, the worse the prognosis will be [9].

### 2.5. Statistical Methods

SPSS 22.0 software was used to process the abovementioned data, the count data was expressed as percentage (%), and the *χ*^2^ test was performed between groups; the measurement data that met normality and homogeneity of variance were expressed as mean ± standard deviation ($\bar{x}\pm s$), and one-way analysis of variance was used. Comparison between groups was carried out by using the Student-Newman-Keuls method. A receiver operating characteristic (ROC) curve was used to analyze the predictive value of NLR and RDW in the prognosis of emergency sepsis patients. Multivariate Logistic regression analysis was used to analyze the independent risk factors affecting the prognosis of emergency department sepsis patients, *P* < 0.05 was considered statistically significant.

## 3. Results

### 3.1. Prognosis Analysis of Emergency Patients with Sepsis

Among the 63 emergency department sepsis patients, 41 (65.08%) patients survived and were included in the survival group; 22 (34.92%) patients died and were included in the death group.

### 3.2. Univariate Analysis of the Prognosis of Emergency Patients with Sepsis

There was no significant difference in gender, proportion of underlying diseases and age, BMI index, blood lipid index, coagulation index, Hct, HB, and PLT levels between the two groups (*P* > 0.05); the NLR, RDW, and SOFA scores in the death group were higher than those in the survival group (*P* < 0.05), as shown in Table 1.

### 3.3. Multivariate Logistic Regression Analysis on the Prognosis of Emergency Patients with Sepsis

The prognosis of emergency patients with sepsis was used as the dependent variable, the abovementioned univariate factors with differences were used as independent variables and included in the logistic regression analysis model, quantitative assignment was performed, as shown in Table 2.

Multivariate logistic regression analysis showed that NLR and RDW were independent risk factors affecting the prognosis of emergency patients with sepsis (*P* < 0.05), as shown in Table 3.

### 3.4. The Predictive Value of NLR and RDW on the Prognosis of Emergency Patients with Sepsis

**Figure 1:**
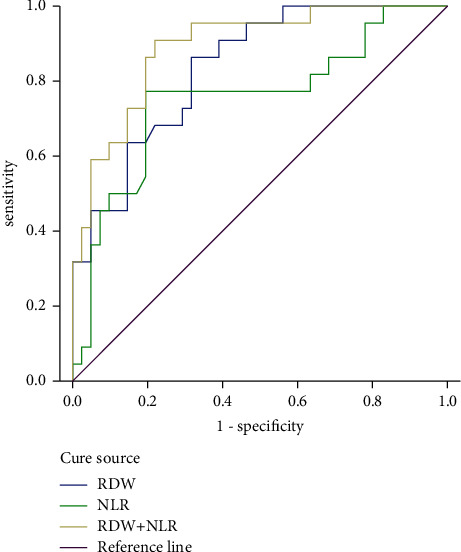
ROC curve of prognostic value of NLR, RDW, and the combination of the two indexes in emergency sepsis patients.

Through ROC curve analysis, the area under the curve of NLR and RDW predicting the prognosis of patients with emergency sepsis was 0.818 and 0.823, respectively, while the area under the curve of the above two indicators combined to diagnose the prognosis of acute sepsis patients was 0.891, as shown in Table 4 and Figure 1.

## 4. Discussion

Sepsis is a common clinical disease of systemic inflammatory response syndrome that is mostly caused by the invasion of pathogenic microorganisms such as bacteria. At present, the clinical treatment of sepsis patients is mainly based on cluster therapy combined with critical care. Although it can help patients improve clinical symptoms and prolong their survival time to a certain extent, the mortality rate of emergency sepsis patients is still high [10, 11]. Studies [12] pointed out that the mortality rate of emergency patients with sepsis is about 36.27%. In this study, the mortality rate of the 63 patients with sepsis in the emergency department accounted for 34.92%. Although there was some deviation from the above report due to the sample size, the difference was not significant. This indicates that the high mortality rate of sepsis in emergencies is still a characteristic worthy of attention. Previous studies have shown that for emergency sepsis, it is of great significance to find quick and correct serum markers to evaluate the prognosis of patients at an early stage and improve the clinical outcome [13]. Both NLR and RDW have been found to be correlated with the prognosis of sepsis patients, and both have been proven to be independent predictors of inpatient mortality in sepsis patients [14]. However, the impact of both on the prognosis of emergency patients with sepsis is still controversial [15]. For this reason, this paper starts a preliminary discussion on this aspect.

It was found in this study that NLR was an independent risk factor affecting the prognosis of emergency department patients with sepsis (*P* < 0.05), which was similar to results described in Pantzaris et.al. reports [16]. This indicates that the NLR level of sepsis patients is closely related to the severity of the disease. NLR is one of the common markers of systemic inflammatory responses in which lymphocytes can eliminate nonspecific inflammation while neutrophils are important cell that responds to nonspecific inflammatory responses and secret destructive enzymes and inflammatory mediators [17]. Sepsis is a systemic inflammatory disease caused by various pathogens. The neutrophils and lymphocytes in peripheral blood are the main effector cells involved in the inflammatory response [18]. After inflammation, cytokines such as cortisol, prolactin, and catecholamine can be increased. At the same time, it leads to the spontaneous apoptosis of lymphocytes attached to the reticuloendothelial cell system, causing a persistent and harmful inflammatory state, delaying the apoptosis of neutrophils and increasing the contentration of neutrophils in the blood. If sepsis is not effectively controlled in time, neutrophils can accumulate in large numbers in the organs and tissues of the patient's body, block the microcirculation, and aggravate the damage, which is not conducive to prognosis. In addition, when bacteria invade the body of emergency patients with emergency sepsis, the expression of neutrophil surface receptors is out of balance, and a large number of functionally immature neutrophils are released into the blood, which will not only increase the number of neutrophils but also stimulate the activation of lymphocytes and migration to inflammatory tissues, reduce lymphocyte apoptosis, and then, increase the degree of functional damage to vital organs, further aggravating the condition of sepsis patients.

It was found in studies that there is a correlation between the occurrence and development of sepsis and the level of RDW [19]. Other studies point out that RDW is an important predictor of death in patients with sepsis [20]. In the results of this study, RDW was an independent risk factors affecting the prognosis of patients with sepsis in the emergency department (*P* < 0.05), and it was confirmed that the level of RDW could reflect the prognosis of patients with sepsis in the emergency department. The reason is that RDW represents the degree of red blood cell dispersion, which can intuitively reflect the heterogeneity of red blood cell volume. Interaction between RDW levels and severity of inflammatory response in emergency sepsis. When sepsis occurs, the systemic inflammatory response can inhibit red blood cell maturation, increase the level of circulating immature reticulocytes, reduce the half-life of red blood cells, increase the heterogeneity of red blood cells, and cause a large number of immature red blood cells to enter the blood, thereby increasing the RDW level. At the same time, the increase of RDW level can lead to iron metabolism disorder, damage the hematopoietic function, because bone marrow suppression, reduce the expression of erythropoietin receptors, and aggravate the inflammatory reaction. This is repeated to form a vicious circle.

In this study, through ROC curve analysis, it was found that both NLR and RDW had certain predictive values for the prognosis of emergency patients with sepsis, and the areas under the curve were 0.818 and 0.823, respectively. At the same time, the combined detection of the two also has a certain predictive value for the prognosis of emergency patients with sepsis.

To sum up, emergency patients with sepsis have a certain risk of poor prognosis. When the NLR and RDW levels of patients increase, it indicates that the patients may die. Therefore, the detection of NLR and RDW levels can timely determine the risk of death and provide a reference for symptomatic treatment. The sample size selected for this study is relatively low, so there are certain limitations. In the future, the sample size can be appropriately expanded for in-depth research to provide objective theoretical support for this field.

## Figures and Tables

**Table 1:** Univariate analysis on those affecting the prognosis of emergency patients with sepsis.

Indicator	Survival group (*n* = 41)	Death group (*n* = 22)	*t/χ* ^2^	*P*
Age (years old)	44.76 ± 5.16	44.69 ± 4.13	0.156	0.876

Gender (male/female)	23/18	19/13	0.052	0.818

*Basic illness* (*n*, %)				
Hypertension	13 (31.71)	7 (31.82)	0.001	0.992
Diabetes	8 (19.51)	2 (9.09)	1.164	0.280
Coronary heart disease	5 (12.19)	3 (13.64)	0.026	0.869
SOFA (score)	3.71 ± 1.12	14.38 ± 2.16	25.906	0.001
BMI (kg/m^2^)	25.74 ± 1.34	26.08 ± 1.21	0.992	0.325

*Blood lipids*				
TC (mmo/l/L)	4.28 ± 0.81	4.21 ± 0.77	0.332	0.740
TG (mmo/l/L)	2.91 ± 0.43	2.88 ± 0.38	0.274	0.784
HDL-C (mmo/l/L)	1.51 ± 0.25	1.48 ± 0.23	0.466	0.642
LDL-C (mmo/l/L)	2.32 ± 0.57	2.29 ± 0.54	0.202	0.840

*Coagulation*				
TT (s)	13.85 ± 2.71	13.98 ± 2.45	0.187	0.851
APTT (s)	28.96 ± 2.15	27.98 ± 2.84	1.538	0.129
PT (s)	12.29 ± 1.98	12.42 ± 1.76	0.257	0.797
DD (*μ*g/L)	175.23 ± 9.48	177.18 ± 9.89	0.766	0.446
HB (g/L)	126.53 ± 20.11	125.96 ± 21.74	0.104	0.917
Hct (%)	0.32 ± 0.08	0.35 ± 0.09	1.358	0.179
PLT (×10^9^/L)	214.33 ± 20.15	217.84 ± 19.86	0.662	0.510
RDW (%)	11.33 ± 2.51	17.42 ± 2.68	8.967	0.001
NLR	11.45 ± 1.52	18.96 ± 2.03	16.590	0.001

**Table 2:** Quantitative assignment table.

Variable	Quantitative assignment
NLR	<16.93 = 0, ≥16.93 = 1
RDW	<16.00% = 0, ≥16.00% = 1
SOFA score	<9 scores = 0, ≥9 scores = 1
Prognosis	Survival = 0, death = 1

**Table 3:** Multivariate logistic regression analysis on those affecting the prognosis of emergency patients with sepsis.

Variable	*β*	*S.E.*	*Wald*	*P*	*OR*	95% CI
NLR	0.308	0.102	9.018	0.002	1.361	1.113∼1.664
RDW	0.354	0.133	7.091	0.007	1.425	1.098∼1.849
SOFA score	0.449	0.953	0.637	0.619	1.567	0.242∼10.146

**Table 4:** Predictive value of NLR and RDW on the prognosis of emergency patients with sepsis.

Indicator	Area under the curve	Standard error	*P* value	95% CI	Best cutoff value	Sensitivity	Specificity
NLR	0.818	0.063	0.001	0.694∼0.941	16.935	0.82	0.69
RDW	0.823	0.056	0.001	0.713∼0.933	16.000	0.82	0.60
NLR + RDW	0.891	0.072	0.001	0.507∼0.790	—	0.81	0.51

## Data Availability

The data used in this study can be obtained from the corresponding author upon reasonable request.

## References

[1] Olejarova M., Dobisova A., Suchankova M. (2019). Vitamin D deficiency—a potential risk factor for sepsis development, correlation with inflammatory markers, SOFA score and higher early mortality risk in sepsis. *Bratislava Medical Journal*.

[2] Madushani R. W. M. A., Patel V., Bihorac A. (2022). Early biomarker signatures in surgical sepsis. *Journal of Surgical Research*.

[3] Hammond N. E., Kumar A., Venkatesh B. (2022). Estimates of sepsis prevalence and outcomes in adult patients in the ICU in India: a cross-sectional study. *Chest*.

[4] Kim J., Kim K., Lee H., Ahn S. (2019). Epidemiology of sepsis in Korea: a population-based study of incidence, mortality, cost and risk factors for death in sepsis. *Clinical and Experimental Emergency Medicine*.

[5] Kopczynska M., Sharif B., Cleaver S. (2018). Red-flag sepsis and SOFA identifies different patient population at risk of sepsis-related deaths on the general ward. *Medicine (Baltimore)*.

[6] Arvaniti K., Dimopoulos G., Blot S. (2022). Epidemiology and age-related mortality in critically ill patients with intra-abdominal infection or sepsis: an international cohort study. *International Journal of Antimicrobial Agents*.

[7] Shankar-Hari M., Phillips G. S., Levy M. L. (2016). Developing a new definition and assessing new clinical criteria for septic shock: for the third international consensus definitions for sepsis and septic shock (sepsis-3). *JAMA*.

[8] Chen F. C., Kung C. T., Cheng H. H. (2019). Quick sepsis-related organ failure assessment predicts 72 h mortality in patients with suspected infection. *European Journal of Emergency Medicine*.

[9] Brakenridge S. C., Efron P. A., Cox M. C. (2019). Current epidemiology of surgical sepsis: discordance between inpatient mortality and 1-year outcomes. *Annals of Surgery*.

[10] Huang C. T., Ruan S. Y., Tsai Y. J., Ku S. C., Yu C. J. (2019). Clinical trajectories and causes of death in septic patients with a low apache II score. *Journal of Clinical Medicine*.

[11] Meyer N., Harhay M. O., Small D. S. (2018). Temporal trends in incidence, sepsis-related mortality, and hospital-based acute care after sepsis. *Critical Care Medicine*.

[12] Souza D. C., Martin J. G., Machado F. R. (2021). The epidemiology of sepsis in paediatric intensive care units in Brazil (the sepsis prevalence assessment database in pediatric population, spread ped): an observational study. *The Lancet Child & Adolescent Health*.

[13] Brooks D., Polubothu P., Young D., Booth M., Smith A. (2018). Sepsis caused by bloodstream infection in patients in the intensive care unit: the impact of inactive empiric antimicrobial therapy on outcome. *Journal of Hospital Infection*.

[14] Khosrojerdi A., Soudi S., Hosseini A. Z., Eshghi F., Shafiee A., Hashemi S. M. (2021). Immunomodulatory and therapeutic effects of mesenchymal stem cells on organ dysfunction in sepsis. *Shock*.

[15] Akinosoglou K., Kapsokosta G., Gogos C. (2020). Diabetes on sepsis outcomes in non-ICU patients: a cohort study and review of the literature. *Journal of Diabetes and Its Complications*.

[16] Pantzaris N. D., Platanaki C., Pierrako C., Karamouzos V., Velissaris D. (2018). Neutrophil-to-lymphocyte ratio relation to sepsis severity scores and inflammatory biomarkers in patients with community-acquired pneumonia: a case series. *Journal of Translational Internal Medicine*.

[17] Sano H., Kobayashi R., Iguchi A. (2017). Risk factors for sepsis-related death in children and adolescents with hematologic and malignant diseases. *Journal of Microbiology, Immunology, and Infection*.

[18] Simpson G., Saunders R., Wilson J., Magee C. (2018). The role of the neutrophil:lymphocyte ratio (NLR) and the CRP:albumin ratio (CAR) in predicting mortality following emergency laparotomy in the over 80 age group. *European Journal of Trauma and Emergency Surgery*.

[19] Umemura K., Ogura H., Gando S. (2020). Current spectrum of causative pathogens in sepsis: a prospective nationwide cohort study in Japan. *International Journal of Infectious Diseases*.

[20] Berg M. V. D., Beuningen F. E. V., Bouma H. R. (2022). Hospital-related costs of sepsis around the world: a systematic review exploring the economic burden of sepsis. *Journal of Critical Care*.

